# The Transformative Potential of AI in Ultrasound for Facial Aesthetics

**DOI:** 10.1111/jocd.16691

**Published:** 2024-11-25

**Authors:** Diala Haykal, Hugues Cartier, Kyuho Yi, Ximena Wortsman

**Affiliations:** ^1^ Centre Laser Palaiseau Palaiseau France; ^2^ Centre Médical Saint Jean Arras France; ^3^ Department of Aesthetics Maylin Clinic (Apgujeong) Seoul South Korea; ^4^ Department of Dermatology, Faculty of Medicine Universidad de Chile Santiago Chile; ^5^ Department of Dermatology Pontificia Universidad Catolica de Chile Santiago Chile; ^6^ Institute for Diagnostic Imaging and Research of the Skin and Soft Tissues Santiago Chile

## Abstract

**Background:**

The integration of artificial intelligence (AI) and ultrasound (US) technology is reshaping facial aesthetics, providing enhanced diagnostic precision, procedural safety, and personalized patient care. The variability in US imaging, stemming from patient anatomy, operator skills, and equipment diversity, poses challenges in achieving consistent and accurate outcomes. AI addresses these limitations by standardizing imaging protocols, automating image analysis, and supporting real‐time decision‐making.

**Objective:**

To explore the applications of AI‐enhanced US in facial aesthetics, focusing on its potential to improve diagnostic accuracy, procedural safety, and personalized treatments while identifying future prospects and challenges.

**Methods:**

A comprehensive review of current literature and advancements was conducted, examining the integration of AI with US in facial aesthetics. Key areas of focus included AI algorithms for image enhancement, real‐time guidance during procedures, postprocedure assessment, personalized treatment planning, and workflow optimization.

**Results:**

AI‐enhanced US significantly improved diagnostic accuracy by automating the identification of critical anatomical structures and reducing operator variability. Real‐time guidance during procedures enhanced safety, reducing complications such as vascular occlusion and nerve damage. Postprocedure assessments facilitated early detection of complications and improved patient outcomes. Personalized treatment plans tailored to individual anatomy and clinical needs resulted in higher patient satisfaction. Additionally, AI optimized workflow efficiency through seamless integration with electronic health records and advanced training simulators.

**Conclusion:**

The integration of AI and US technology represents a transformative advancement in facial aesthetics. By enhancing precision, safety, and personalization, AI‐powered US sets new benchmarks in diagnostic accuracy and treatment outcomes. Despite challenges related to data diversity, ethical considerations, and training, this synergy holds immense potential to revolutionize the field, offering improved outcomes and satisfaction for practitioners and patients alike. Further research and innovation are essential to fully realize the benefits of this technology.

## 1 Introduction

In the ever‐evolving field of facial aesthetics, technology continuously pushes the boundaries of what is possible. One of the most promising advancements lies at the intersection of artificial intelligence (AI) and ultrasound (US) technology. As facial aesthetics procedures become more sophisticated, the integration of AI into US could revolutionize the industry, offering unprecedented precision, safety, and outcomes [1]. US examinations in medical practice are inherently complex and variable due to a range of factors. These include variations in patient anatomy, movement artifacts, and physiology; operator skills and experience in imaging techniques and adjusting parameters. Another aspect of this complexity is owing to the diverse span of specialties in clinical indications, each with unique imaging requirements and diagnostic challenges [2]. Technological differences in US equipment and transducer types also contribute to variability in imaging approaches. Addressing these challenges, AI holds promise in standardizing protocols, automating image analysis, and providing tailored clinical decision support to improve US examinations' reliability, efficiency, and accuracy across various clinical settings [3]. AI algorithms are leveraged to significantly improve the quality of US images by enhancing contrast and refining details, thereby facilitating clearer visualization of anatomical structures. Moreover, these algorithms enable automated analysis of US images that can support the detection and measurement of abnormalities or lesions [4]. This automation not only saves time for healthcare professionals but also enhances diagnostic accuracy by providing objective assessments based on image patterns and features [5, 6]. Furthermore, AI‐driven computer‐aided diagnosis systems play a pivotal role in supporting radiologists and clinicians. These systems provide real‐time feedback and assist in generating potential diagnoses, thereby augmenting decision‐making processes. Additionally, AI helps optimize workflow management within healthcare settings by prioritizing urgent cases, organizing image data efficiently, and integrating seamlessly with electronic health records (EHR) systems. In the realm of personalized medicine, AI algorithms analyze patient‐specific data derived from US images and clinical information, contributing to tailored treatment plans and, therefore improved patient outcomes.

This article underscores how AI in US revolutionizes medical imaging in facial aesthetics by enhancing diagnostic capabilities, streamlining workflows, and supporting personalized patient care, thereby advancing the field of facial aesthetics diagnostics and treatment.

## 2 Enhancing Diagnostic Accuracy

Through real‐time imaging, US helps practitioners visualize underlying structures, consequently, it has long been a staple in medical diagnostics. In facial aesthetics, accurate mapping of facial anatomy is crucial [7]. AI can significantly enhance this capability by analyzing US images with a level of precision and speed beyond human capacity. Machine learning algorithms are trained to recognize and delineate anatomical structures, such as nerves, blood vessels, and fat compartments, with remarkable accuracy. This can reduce the risk of complications during procedures like filler injections, where precise placement is essential to avoid adverse effects. AI‐based US tools use algorithms to analyze images in real time and produce maps of facial anatomy. This allows practitioners to identify the optimal injection sites while avoiding critical structures such as blood vessels and nerves. The result can significantly reduce complications such as vascular occlusion and nerve damage. Similarly, patients show higher satisfaction rates due to the more natural and symmetrical outcomes achieved [8, 9, 10, 11]. To this end, Muse and Topol explored the transformative potential of AI in improving US image acquisition. They demonstrated how AI standardizes and optimizes US scans through machine learning algorithms that adjust imaging parameters such as depth, gain, and focus. By adapting to patient‐specific characteristics, AI‐guided US reduces operator variability and enhances consistency across healthcare settings. This improves diagnostic accuracy and workflow efficiency by reducing the need for repeated scans and speeding up diagnosis and treatment. The integration of AI in US image capture supports digital health innovation, advancing personalized medicine, optimizing resource use, and improving patient outcomes [4]. Muse and Topol advocate for continued research and implementation of AI‐powered US solutions to enhance diagnostic precision and healthcare delivery [3].

As a more specific example, a prebuilt neural network architecture (EfficientNet B4) in a commercial AI platform studied 235 color Doppler images of both benign and malignant cutaneous lesions. The algorithm sensitivity, specificity, and predictive positive and negative values for the validation algorithm were 0.8, 0.86, 0.86, and 0.8, respectively, for malignancy diagnosis. The statistical results were not significantly different from the dermatologist's evaluation [12]. The use of such algorithms in analyzing cutaneous lesions is promising due to its high level of accuracy in diagnosing malignancy with sensitivity and specificity comparable to those of dermatologists. This suggests two significant advantages: first, the algorithm can assist doctors by providing a second opinion, thereby reducing the likelihood of diagnostic errors; this can be particularly valuable in busy clinical settings or in areas with limited access to dermatologists, ensuring more consistent and accurate diagnoses; second, in the future, such algorithms could handle routine evaluations, freeing up doctors to focus on more complex cases. This could lead to more efficient use of medical resources and potentially lower healthcare costs.

Overall, the integration of AI in dermatology has the potential to enhance diagnostic accuracy and efficiency, benefiting both patients and healthcare providers.

## 3 Personalized Treatment Plans

The ability of AI to process vast amounts of data quickly facilitates the creation of highly personalized treatment plans. By integrating patient‐specific data, such as facial anatomy, skin type, and previous treatment history, AI helps practitioners tailor interventions that are uniquely suited to each individual. This personalized approach can enhance the effectiveness of treatments, improve patient satisfaction, and reduce the likelihood of adverse reactions. AI‐driven US technology has been instrumental in developing personalized treatment plans for patients seeking facial rejuvenation. By analyzing each patient's unique facial structure and skin condition, the AI system could recommend tailored intervention strategies. Kim et al. underscore the critical importance of developing tailored AI‐driven plans for integrating AI into medical ultrasonography. They emphasize that AI technologies can be customized to meet specific clinical needs and challenges in US imaging, such as optimizing image acquisition parameters based on patient demographics, anatomical variations, and clinical indications. Tailored AI plans are crucial for enhancing diagnostic accuracy by addressing the unique complexities and variability inherent in US examinations [13]. For instance, in treatments involving radiofrequency or US‐based skin tightening, the AI system adjusted the intensity and duration of energy delivery based on real‐time feedback from US imaging. This customization led to more effective treatments with faster recovery times and minimal side effects, enhancing overall patient experience and outcomes. Furthermore, personalized AI applications can support healthcare providers in making more informed decisions, improving patient outcomes, and streamlining workflow efficiency.

## 4 Enhancing Safety in Surgical Procedures

In facial reconstructive surgery, precision is paramount for decreasing errors leading to irreversible damage. AI algorithms can help in detailed preoperative planning by creating 3D models of the patient's facial anatomy from US data [14]. During surgery, the real‐time guidance provided by AI‐enhanced US ensures accurate dissection planes and implant placements, significantly reducing operative time and improving surgical outcomes [15]. In the postoperative phase, AI‐enhanced US plays an important role by detecting complications more reliably [16].

## 5 Real‐Time Guidance and Monitoring

During facial aesthetic procedures, real‐time guidance is crucial. AI‐enhanced US provides practitioners with live feedback, ensuring that injections or other interventions are being performed precisely as planned. This real‐time monitoring alerts practitioners to any deviations from the optimal path, allowing for immediate adjustments. For instance, if an AI system detects that a needle is approaching a critical structure like a nerve or blood vessel, it can provide instant warnings, enhancing the safety and precision of the procedure.

## 6 Postprocedure Assessment and Follow‐Up

The benefits of AI in US extend beyond the procedure itself. Postprocedure assessment is a critical component of facial aesthetics, ensuring that the desired outcomes have been achieved and identifying any potential complications early on [17]. AI automates the analysis of postprocedure US images, comparing them with preprocedure baselines and expected outcomes. This also helps practitioners quickly identify any issues, such as filler migration or unexpected tissue reactions, and take appropriate corrective measures instantly. Postoperative US assessments, guided by AI, help in the early detection of complications such as hematomas or seromas, allowing for prompt intervention. In various studies, AI‐enhanced US was useful for monitoring patients after various aesthetic treatments, including fat grafting and thread lifts. The AI system might help compare postprocedure US images with baseline images to detect subtle changes and potential complications in advance. This proactive approach enabled timely interventions, improving patient safety and satisfaction. For example, in cases where filler migration was detected early, corrective measures were taken before any aesthetic compromise occurred [2, 8, 9, 18]. Additionally, AI can detect vascular occlusion and involved vessels, guiding the injection of hyaluronidase, and identify nodules and granulomas, facilitating their treatment [19].

## 7 Electronic Health Records

AI has the potential to revolutionize EHRs by enhancing efficiency, accuracy, and accessibility across healthcare settings. AI‐powered natural language processing can extract and analyze unstructured data from clinical notes, improving data integration and documentation accurately [20]. Clinical decision support systems powered by AI can offer real‐time guidance based on patient‐specific data and clinical guidelines, improving diagnostic rigor and patient safety. AI‐driven predictive analytics enable healthcare organizations to proactively manage population health, identify trends in patient outcomes, and optimize resource allocation. In medical imaging, AI algorithms can analyze images stored in EHRs, aiding diagnosis by detecting abnormalities and prioritizing urgent cases [21]. Moreover, AI enhances workflow efficiency by automating administrative tasks and supports personalized medicine by analyzing patient data to tailor treatment plans. What is more, AI alleviates the burden associated with EHRs by streamlining documentation processes through voice‐to‐text transcription, automated summarization, and clinical documentation improvement tools [22]. These AI solutions reduce physicians' time on administrative tasks such as data entry and note taking, thereby allowing them to focus more on direct patient care [23]. Virtual scribes and automated workflow further enhance efficiency by handling routine documentation and order processing, while AI algorithms improve the meticulousness and completeness of medical records by identifying errors and inconsistencies [24]. Additionally, AI can help manage alerts and notifications to prevent alert fatigue and optimize time management, ultimately reducing physician burnout, enhancing job satisfaction, and improving patient care quality. Continuous learning capabilities allow AI within EHRs to evolve with new data inputs and medical knowledge, improving over time to deliver more effective healthcare solutions.

## 8 Data Dependency and Diagnostic Limitations

The integration of AI in dermatological US hinges significantly on the quality and diversity of its training data. AI algorithms require large, varied datasets to function accurately across different populations and clinical settings. Given the variations in skin types, US equipment, and diagnostic standards globally, algorithms trained on limited or homogenous data are at risk of producing biased or unreliable results. This challenge highlights the critical need for diverse, representative datasets to prevent inaccuracies that could impact patient safety [25]. Furthermore, while AI demonstrates impressive capabilities in pattern recognition, it lacks the contextual understanding that is essential in dermatologic diagnostics. AI systems cannot fully consider factors like patient history, lifestyle, or environmental influences, elements that are crucial in developing comprehensive assessments in dermatology. Without this contextual insight, there is a risk of misdiagnosis or unnecessary interventions if clinicians rely solely on AI outputs. Thus, AI should be viewed as an adjunctive tool, augmenting rather than replacing the clinician's judgment.

## 9 Training and Education

Incorporating AI in US also holds significant potential for training and education in facial aesthetics for physicians. AI‐driven simulators can provide practitioners with realistic, hands‐on training experiences, helping them hone their skills in a controlled, risk‐free environment [26]. By analyzing trainees' performance and providing instant feedback, these AI systems can accelerate the learning curve and ensure that practitioners are well prepared for real‐world scenarios [27]. Aesthetic training academies have adopted AI‐enhanced US simulators to improve the skill sets of budding practitioners. These simulators, powered by sophisticated AI algorithms, provide realistic training scenarios where trainees can practice various procedures such as botulinum toxin injections and filler placements [28, 29]. The AI system offers real‐time feedback, highlighting mistakes and suggesting corrections. This hands‐on approach has proven to accelerate the learning curve, with trainees demonstrating improved technique and confidence in actual clinical settings [15, 19].

## 10 Ethical and Legal Concerns

Beyond technical limitations, the adoption of AI in dermatological US introduces ethical and legal complexities. In the event of a misdiagnosis, questions arise regarding liability, whether responsibility should fall on healthcare providers, AI developers, or the institutions deploying these systems. This ambiguity creates a barrier to clinical adoption, as practitioners may hesitate to trust or implement AI without clear regulatory guidance. Additionally, a lack of transparency in AI decision‐making processes may erode patient trust, as clinicians and patients alike require assurance of accountability and reliability. Establishing robust ethical guidelines and regulatory standards will be essential for integrating AI responsibly in dermatologic applications [30, 31]. By addressing these ethical and legal challenges, the field can advance toward a safe and trusted use of AI, ensuring that it benefits both practitioners and patients in clinical dermatology.

## 11 Future Prospects and Challenges

In the future, thanks to AI‐based US detection for filler products, complications like distinguishing between hematomas and abscesses and identifying parotid duct injuries will become essential tools for diagnosing side effects in facial aesthetics [32]. However, despite the immense potential of AI in US for facial aesthetics, there are significant challenges to address. Given the sensitive nature of patient information, data privacy and security are paramount. Ensuring that AI systems are transparent and explainable is also crucial, as practitioners must understand how decisions are being made to maintain trust and accountability. Standardization of the acquisition of images and better databases on the US patterns and their anatomical variants are critical. Moreover, integrating AI into clinical practice requires significant investment in training and infrastructure [31]. Despite these challenges, the future looks promising. As AI technology continues to advance, its integration with US could set new standards in facial aesthetics, offering safer, more precise, and personalized treatments. For patients and practitioners alike, this could mean better outcomes, fewer complications, and a higher level of satisfaction [33].

## 12 Conclusion

In conclusion, the convergence of AI and US in facial aesthetics represents a groundbreaking development. By enhancing diagnostic accuracy, enabling personalized treatment plans, providing real‐time guidance, and improving postprocedure assessment, AI has the potential to transform the field. As we navigate the challenges and embrace the opportunities, the future of facial aesthetics looks brighter than ever. These real‐world applications underscore the significant impact that AI‐enhanced US can have on the practice of facial aesthetics. By improving diagnostic accuracy, enabling personalized treatments, enhancing procedural safety, and facilitating comprehensive training, AI is not just an adjunct but a pivotal component in advancing facial aesthetics. As more clinics, companies, and practitioners adopt this technology, we can expect to see continued improvements in patient outcomes and satisfaction, setting new standards in the field. The future of facial aesthetics is here, and the synergy of AI and US technology should power it. By integrating AI into US technology, the field of facial aesthetics can achieve new heights of precision, safety, and personalized care. This synergy's transformative potential holds promise for practitioners and patients, heralding a new era of innovation and excellence in aesthetic medicine.

## Author Contributions

D.H. wrote the manuscript, and H.C., K.Y., and X.W. reviewed it.

## Ethics Statement

The authors have nothing to report.

## Consent

The authors have nothing to report.

## Conflicts of Interest

The authors declare no conflicts of interest.

## Data Availability

The data that support the findings of this study are available on references part. All information is included in the manuscript.

## References

[1] P. J. Velthuis , O. Jansen , L. W. Schelke , et al., “A Guide to Doppler Ultrasound Analysis of the Face in Cosmetic Medicine. Part 1: Standard Positions,” Aesthetic Surgery Journal 41, no. 11 (2021): NP1621–NP1632, 10.1093/asj/sjaa410. 33954581

[2] X. Wortsman , “Practical Applications of Ultrasound in Dermatology,” Clinics in Dermatology 39, no. 4 (2021): 605–623, 10.1016/j.clindermatol.2021.03.007. 34809766

[3] E. D. Muse and E. J. Topol , “Guiding Ultrasound Image Capture With Artificial Intelligence,” Lancet 396, no. 10253 (2020): 749, 10.1016/S0140-6736(20)31875-4. 32919501

[4] D. Haykal , H. Cartier , D. du Crest , H. Galadari , M. Landau , and A. Haddad , “What Happens When Simulations Get Real and Cosmetic Dermatology Goes Virtual?,” Journal of Cosmetic Dermatology 23 (2023): 2682–2684, 10.1111/jocd.15888. 37353976

[5] R. Tenajas , D. Miraut , C. I. Illana , R. Alonso‐Gonzalez , F. Arias‐Valcayo , and J. L. Herraiz , “Recent Advances in Artificial Intelligence‐Assisted Ultrasound Scanning,” Applied Sciences 13, no. 6 (2023): 3693, 10.3390/app13063693.

[6] X. Liang , X. Yang , S. Yin , et al., “Artificial Intelligence in Plastic Surgery: Applications and Challenges,” Aesthetic Plastic Surgery 45, no. 2 (2021): 784–790, 10.1007/s00266-019-01592-2. 31897624

[7] D. Haykal , H. Cartier , M. Benzaquen , G. Damiani , and S. M. Habib , “The Growing Importance of Ultrasonography in Cosmetic Dermatology: An Update After the 23rd IMCAS Annual World Congress (2022),” Journal of Cosmetic Dermatology 22, no. 1 (2023): 222–225, 10.1111/jocd.15503. 36374262

[8] L. W. Schelke , D. Cassuto , P. Velthuis , and X. Wortsman , “Nomenclature Proposal for the Sonographic Description and Reporting of Soft Tissue Fillers,” Journal of Cosmetic Dermatology 19, no. 2 (2020): 282–288, 10.1111/jocd.13127. 31456355

[9] L. W. Schelke , P. Velthuis , J. Kadouch , and A. Swift , “Early Ultrasound for Diagnosis and Treatment of Vascular Adverse Events With Hyaluronic Acid Fillers,” Journal of the American Academy of Dermatology S0190‐9622, no. 19 (2019): 32392–32398, 10.1016/j.jaad.2019.07.032. 31325548

[10] L. Schelke , P. J. Velthuis , N. Lowry , et al., “The Mobility of the Superficial and Deep Midfacial Fat Compartments: An Ultrasound‐Based Investigation,” Journal of Cosmetic Dermatology 20, no. 12 (2021): 3849–3856, 10.1111/jocd.14374. 34365716

[11] L. Schelke , T. Decates , J. Kadouch , and P. Velthuis , “Incidence of Vascular Obstruction After Filler Injections,” Aesthetic Surgery Journal 40, no. 8 (2020): NP457–NP460, 10.1093/asj/sjaa086. 32538425 PMC7357869

[12] A. Laverde‐Saad , A. Jfri , R. García , et al., “Discriminative Deep Learning Based Benignity/Malignancy Diagnosis of Dermatologic Ultrasound Skin Lesions With Pretrained Artificial Intelligence Architecture,” Skin Research and Technology 28, no. 1 (2021): 35–39, 10.1111/srt.13086. 34420233 PMC9907620

[13] Y. H. Kim , “Artificial Intelligence in Medical Ultrasonography: Driving on an Unpaved Road,” Ultrasonography 40, no. 3 (2021): 313–317, 10.14366/usg.21031. 34053212 PMC8217795

[14] J. L. Sparling , B. Hong Mershon , and J. Abraham , “Perioperative Handoff Enhancement Opportunities Through Technology and Artificial Intelligence: A Narrative Review,” Joint Commission Journal on Quality and Patient Safety 49, no. 8 (2023): 410–421, 10.1016/j.jcjq.2023.03.009. 37202263

[15] E. Jacobs , B. Wainman , and J. Bowness , “Applying Artificial Intelligence to the Use of Ultrasound as an Educational Tool: A Focus on Ultrasound‐Guided Regional Anesthesia,” Anatomical Sciences Education 17, no. 5 (2024): 919–925, 10.1002/ase.2266. 36880869

[16] D. Del Vecchio , M. J. Stein , E. Dayan , J. Marte , and S. Theodorou , “Nanotechnology and Artificial Intelligence: An Emerging Paradigm for Postoperative Patient Care,” Aesthetic Surgery Journal 43, no. 7 (2023): 748–757, 10.1093/asj/sjad071. 36944499

[17] D. Haykal , “Unleashing the Power of Biosensors and AI in Dermatology,” Aesthetic Surgery Journal. Open Forum 6 (2024): ojae030, 10.1093/asjof/ojae030. 38756357 PMC11097204

[18] J. Czajkowska , J. Juszczyk , M. N. Bugdol , et al., “High‐Frequency Ultrasound in Anti‐Aging Skin Therapy Monitoring,” Scientific Reports 13, no. 1 (2023): 17799, 10.1038/s41598-023-45126-y. 37853086 PMC10584894

[19] K. H. Yi , “Where and When to Use Ultrasonography in Botulinum Neurotoxin, Fillers, and Threading Procedures?,” Journal of Cosmetic Dermatology 23, no. 3 (2024): 773–776, 10.1111/jocd.16064. 37969045

[20] S. Kataria and V. Ravindran , “Electronic Health Records: A Critical Appraisal of Strengths and Limitations,” Journal of the Royal College of Physicians of Edinburgh 50, no. 3 (2020): 262–268, 10.4997/JRCPE.2020.309. 32936099

[21] X. Yang , A. Chen , N. PourNejatian , et al., “A Large Language Model for Electronic Health Records,” npj Digital Medicine 5, no. 1 (2022): 1–9, 10.1038/s41746-022-00742-2. 36572766 PMC9792464

[22] M. Milne‐Ives , C. de Cock , E. Lim , et al., “The Effectiveness of Artificial Intelligence Conversational Agents in Health Care: Systematic Review,” Journal of Medical Internet Research 22, no. 10 (2020): e20346, 10.2196/20346. 33090118 PMC7644372

[23] Massachusetts Institute of Technology , “Toward a Smarter Electronic Health Record,” 2021, https://news.mit.edu/2021/medknowts‐electronic‐health‐record‐0923.

[24] A. Alanazi , “Clinicians' Views on Using Artificial Intelligence in Healthcare: Opportunities, Challenges, and Beyond,” Cureus 15, no. 9 (2023): e45255, 10.7759/cureus.45255. 37842420 PMC10576621

[25] R. Daneshjou and H. Kittler , “Empowering the Next Generation of Artificial Intelligence in Dermatology: The Datasets and Benchmarks Track of the Journal of Investigative Dermatology,” Journal of Investigative Dermatology 144, no. 3 (2024): 437–438, 10.1016/j.jid.2023.11.011. 38103828

[26] D. Haykal , B. Ascher , H. Cartier , and M. Gold , “Exploring the Landscape of AI Adoption in Cosmetic Medicine and Surgery: Insights From the 25th IMCAS Congress (International Master Course in Aging Science),” Journal of Cosmetic Dermatology 23, no. 8 (2024): 2673–2675, 10.1111/jocd.16316. 38715383

[27] C. Edwards , C. Chamunyonga , B. Searle , and T. Reddan , “The Application of Artificial Intelligence in the Sonography Profession: Professional and Educational Considerations,” Ultrasound 30, no. 4 (2022): 273–282, 10.1177/1742271X211072473. 36969531 PMC10034654

[28] Cutaneous Facial Ultrasound , “Cutaneous,” accessed July 27, 2024, https://cutaneous.nl/.

[29] Think‐In by Dr Benjamin Ascher , “A Luminary in Aging Science From the Heart of Paris,” accessed July 27, 2024, https://thinkin.fr/.

[30] T. E. Sangers , H. Kittler , A. Blum , et al., “Position Statement of the EADV Artificial Intelligence (AI) Task Force on AI‐Assisted Smartphone Apps and Web‐Based Services for Skin Disease,” Journal of the European Academy of Dermatology and Venereology: JEADV 27 (2023): 22–30, 10.1111/jdv.19521. 37766502

[31] S. H. Park , “Artificial Intelligence for Ultrasonography: Unique Opportunities and Challenges,” Ultrasonography 40, no. 1 (2021): 3–6, 10.14366/usg.20078. 33227844 PMC7758099

[32] K. O'Rourke , N. Kibbee , and A. Stubbs , “Ultrasound for the Evaluation of Skin and Soft Tissue Infections,” Missouri Medicine 112, no. 3 (2015): 202–205. 26168591 PMC6170135

[33] H. Zhang , Z. Meng , J. Ru , Y. Meng , and K. Wang , “Application and Prospects of AI‐Based Radiomics in Ultrasound Diagnosis,” Visual Computing for Industry, Biomedicine, and Art 6, no. 1 (2023): 20, 10.1186/s42492-023-00147-2. 37828411 PMC10570254

